# A review on deep learning MRI reconstruction without fully sampled k-space

**DOI:** 10.1186/s12880-021-00727-9

**Published:** 2021-12-24

**Authors:** Gushan Zeng, Yi Guo, Jiaying Zhan, Zi Wang, Zongying Lai, Xiaofeng Du, Xiaobo Qu, Di Guo

**Affiliations:** 1 School of Computer and Information Engineering, Fujian Engineering Research Center for Medical Data Mining and Application, Xiamen University of Technology, Xiamen, China; 2 Department of Electronic Science, National Institute for Data Science in Health and Medicine, Xiamen University, Xiamen, China; 3 School of Information Engineering, Jimei University, Xiamen, China

## Abstract

**Background:**

Magnetic resonance imaging (MRI) is an effective auxiliary diagnostic method in clinical medicine, but it has always suffered from the problem of long acquisition time. Compressed sensing and parallel imaging are two common techniques to accelerate MRI reconstruction. Recently, deep learning provides a new direction for MRI, while most of them require a large number of data pairs for training. However, there are many scenarios where fully sampled k-space data cannot be obtained, which will seriously hinder the application of supervised learning. Therefore, deep learning without fully sampled data is indispensable.

**Main text:**

In this review, we first introduce the forward model of MRI as a classic inverse problem, and briefly discuss the connection of traditional iterative methods to deep learning. Next, we will explain how to train reconstruction network without fully sampled data from the perspective of obtaining prior information.

**Conclusion:**

Although the reviewed methods are used for MRI reconstruction, they can also be extended to other areas where ground-truth is not available. Furthermore, we may anticipate that the combination of traditional methods and deep learning will produce better reconstruction results.

## Introduction

Magnetic resonance imaging (MRI) plays an important role in clinical medicine, and it can visualize human organs and tissues to help follow-up diagnosis. However, MRI has always faced the challenge of long scan time. Before the advent of deep learning, two common methods were used to accelerate MRI, one was compressed sensing (CS) utilizing image compressibility, and another was parallel imaging using redundant information between coils [1–3]. Although these methods have made certain achievements, it still faces the challenges of long iteration time and low acceleration rate.

Recently, deep learning has become a method for accelerating MRI. Compared with traditional methods, it not only improves the quality of reconstructed images but has the advantages of real-time imaging. The quality of images is measured comprehensively by the peak signal-to-noise ratio (PSNR) and mean structure similarity index measure (MSSIM). Higher PSNR means less noise and Higher MSSIM entail better structure similarity with the ground truth. Meanwhile, real-time imaging is important for some clinic applications, for example, deep learning can achieve real-time adaptive magnetic resonance imaging (MRI)-guided radiotherapy by achieving higher acceleration factors to reduce total delays [4] and provide a powerful diagnostic tool for dynamic assessment of wrist function [5]. However, most of them require a large amount of data to perform network learning in a supervised learning manner.

Traditional optimization methods and deep learning in a supervised manner have done a lot of work and related reviews can be found in [6, 7]. Due to physiological constraints such as organ motion or physical constraints such as signal decay, it is difficult, impractical and impossible to obtain fully sampled data. Some researchers try to utilize transfer learning to solve this problem [8], while they still require a small number of fully sampled data to adjust the pre-trained network. Hence, how to perform network learning and image reconstruction in the absence of fully sampled data is an active research topic. Here, the main related methods in deep learning-based MRI reconstruction without fully sampled data are reviewed.

The remainder of this paper is organized as follows. First, we give a brief overview of the traditional reconstruction model, meanwhile, some reconstruction algorithms involved in the following review are roughly discussed. Then deep learning model for MRI reconstruction is illustrated in a supervised manner. Next, we review how to train a reconstruction network without fully sampled data from the perspective of obtaining prior information. Finally, we emphasize the necessity of deep learning reconstruction without fully sampled data and the current challenges and look forward to the future.

## Traditional methods for MRI reconstruction

### Reconstruction model

Reconstructing a high-quality image from under-sampling data is a typical inverse problem. A multi-coil imaging model can be expressed as follows,

1 $${\mathbf{y}} = {\mathbf{Ax}} + {{\varvec{\upeta}}}\;{\text{with}}\;{\mathbf{A}}_{i} { = }{\mathbf{UFS}}_{i} ,$$

where ${\mathbf{x}} \in {\mathbb{C}}^{N}$ is the image to be reconstructed, ${\mathbf{y}} \in {\mathbb{C}}^{M}$ is the noisy measured data $\left( {M < N} \right)$, ${{\varvec{\upeta}}}$ is the noise, and ${\mathbf{A}}$ denotes a measurement operator consisting of a sampling matrix ${\mathbf{U}} \in {\mathbb{R}}^{M \times N}$, Fourier transform operator ${\mathbf{F}}$ and the sensitivity map matrix ${\mathbf{S}}_{i}$ for the *i*th coil. A common reconstruction model is to add a regularization term to constrain its solution space,

2 $$\mathop {\arg \min }\limits_{{\mathbf{x}}} \frac{1}{2}||{\mathbf{y}} - {\mathbf{Ax}}||_{2}^{2} + \lambda \Re ({\mathbf{x}}),$$

where $||{\mathbf{y}} - {\mathbf{Ax}}||_{2}^{2}$ ensures consistency with the measured data, $\Re ({\mathbf{x}})$ is a regularization item, and $\lambda$ is a tradeoff between the data consistency and the regularization terms. In most cases, the difference lies in whether it is single-channel reconstruction or multi-channel [1–3] reconstruction. Meanwhile, multiple regularization items can be chosen such as 2D wavelet [9], total variation (TV) [10], dictionary [11], 3D wavelet [12], 3D k-t sparse, 3D low-rank (k-t SLR) [13]. Some methods are often used to iteratively solve the above optimization problems [6].

Sparsity or low-rankness constraints are often used as priors to reduce the artefacts of the reconstruction image when the acceleration rate is high. Lustig et al. [14, 15] firstly applied compressed sensing to MRI reconstruction and achieved reliable results. Afterwards, researchers found that the key to MRI reconstruction based on compressed sensing lies in the design of the sparse domain, which mainly includes pre-constructed [9, 14–18] or adaptive [12, 19–21] basis and dictionary [11, 22, 23]. Besides, low-rankness methods are mainly used for dynamic and high-dimensional imaging by exploring the relationship between multiple images [24]. The structured low-rankness of k-space is discovered and used for reconstruction [25]. Meanwhile, the low-rankness of the structured matrix is used to jointly reconstruct the image with other aspects, including transform-domain weighted k-space [26–29] and slowly varying image phases [30, 31]. Although good achievements have been achieved, traditional optimization reconstruction methods complete iterations with more time.

### Optimization algorithm

Practical and effective optimization algorithms are essential. A large number of algorithms have been studied to solve various optimization problems. Deep learning networks and optimization iterative reconstruction algorithms still have a certain connection, thus some of them will be reviewed. Here, we only briefly introduce the algorithms that will be involved in the review, including variable-splitting with the quadratic penalty (VSQP) [32], proximal gradient descent (PGD) [33], iterative shrinkage-thresholding algorithm (ISTA) [34], alternate directions method of multipliers (ADMM) [35].

#### VSQP

We use variable-splitting with the quadratic penalty (VSQP) for Eq. (2), the formulation is as follow,

3 $$\begin{aligned} {\mathbf{z}}^{i} & = \mathop {\arg \min }\limits_{{\mathbf{z}}} \mu ||{\mathbf{x}}^{i - 1} - {\mathbf{z}}||_{2}^{2} + \Re ({\mathbf{z}}) \\ & = prox_{\Re } ({\mathbf{x}}^{i - 1} ), \\ \end{aligned}$$

4 $${\mathbf{x}}^{i} = \mathop {\arg \min }\limits_{{\mathbf{x}}} \frac{1}{2}||{\mathbf{y}} - {\mathbf{Ax}}||_{2}^{2} + \mu ||{\mathbf{x}} - {\mathbf{z}}^{i} ||_{2}^{2} ,$$

where ${\mathbf{z}}^{i}$ is the auxiliary intermediate variable, ${\mathbf{x}}^{i}$ is the image to be reconstructed in the *i*th iteration, and $\mu$,$prox_{\Re } ( \cdot )$ is the secondary penalty parameter and proximity operator respectively. In a deep network, this algorithm can be unrolled for a fixed number of iterations as network architecture, Eq. (3) is mainly related to the choice of priors, and can be interpreted as a denoising operation [50], which is executed in the manner of a neural network. Equation (4) depends on the selection of the forward model and corresponds to the data-consistency (DC) layer in the network, which is usually solved by

5 $$({\mathbf{A}}^{H} {\mathbf{A}} + \mu {\mathbf{I}}){\mathbf{x}}^{i} = ({\mathbf{A}}^{H} {\mathbf{y}} + \mu {\mathbf{z}}^{i} ),$$

where $\cdot^{H}$ denotes conjugate transpose, Eq. (5) can be updated by using a conjugate gradient (CG) [36] to avoid the matrix inversion process and cope with multi-coil reconstruction scenarios.

#### PGD

In VSQP, if the following formula is used to update ${\mathbf{x}}^{i}$ instead of Eq. (4), the proximal gradient descent (PGD) is formulated as.

6 $${\mathbf{x}}^{i} = {\mathbf{z}}^{i} + \rho {\mathbf{A}}^{T} ({\mathbf{y}} - {\mathbf{Az}}^{i} ),$$

where $\rho$ is a gradient descent step size. Since the simplest gradient descent is used to update, it often requires more iterations to achieve better results.

#### ISTA

As discussed above, both VSQP and PGD use a network to directly learn the approximation mapping for Eq. (3). Here, an iterative shrinkage-thresholding algorithm (ISTA) is used to guide the completion of Eq. (3). As well known, ISTA comes from solving $l_{1}$ norm problems; however, magnetic resonance images tend to be sparse in a certain domain rather than self-sparse, hence there is no simple closed-form solution. To explain in more detail, we use the following substitution in Eq. (2).

7 $$\Re {(}{\mathbf{x}}{)} = \lambda {||}{\mathbf{\Psi x}}||_{1} ,$$

we can get the final solution by alternately iterating the following sub-problem and equation Eq. (6).

8 $$\begin{aligned} {\mathbf{z}}^{i} &= {{\varvec{\Psi}}}^{H} prox_{\rho \Re } ({\mathbf{\Psi x}}^{i - 1} ) \\ & = \widetilde{\hbar }({\mathbf{x}}^{i - 1} )\Gamma_{\kappa \Re } (\hbar ({\mathbf{x}}^{i - 1} )), \\ \end{aligned}$$

where ${{\varvec{\Psi}}}$ represents a tight frame, $\Gamma_{\kappa } (x)$ denotes shrinkage operator such that

9 $$\Gamma_{\kappa} (x) = {\text{sign(x)}} \cdot {\max\{|{\text{x}}|-\kappa, 0\}},$$

when ISTA meets deep learning, a nonlinear transform operator $\hbar ({\mathbf{x}})$ is used here instead of ${{\varvec{\Psi}}}$ and $\widetilde{\hbar }({\mathbf{x}}^{i - 1} )$ is an inverse operator of $\hbar ({\mathbf{x}}^{i - 1} )$, meanwhile, $\widetilde{\hbar }({\mathbf{x}}^{i - 1} )$ and $\hbar ({\mathbf{x}}^{i - 1} )$ are implemented with a neural network respectively, $\kappa$ is a new parameter that includes $\rho$. More details can be acquired in [37]. Moreover, when tight frame sparsity is enforced, Liu et al.[17] proposed a projected iterative soft-thresholding algorithm (pFISTA) to address the problem that ISTA can not be directly applied to MRI reconstruction, meanwhile, Zhang et al. [38]proved the convergence of pFISTA applied to parallel imaging. Subsequently, Lu et al. [39] constructed pFISTA-SENSE-ResNet network based on pFISTA and achieved better results compared with traditional parallel imaging in terms of MSSIM and PSNR.

#### ADMM

For formula Eq. (2), we can make the following Augmented Lagrangian function by utilizing a new variable

10 $$\mathop {\max }\limits_{{\mathbf{u}}} \mathop {\min }\limits_{{{\mathbf{x}},{\mathbf{v}}}} \frac{1}{2}||{\mathbf{y}} - {\mathbf{Ax}}||_{2}^{2} + \Re ({\mathbf{v}}) + \frac{\nu }{2}||{\mathbf{x}} - {\mathbf{v}} + {\mathbf{u}}||_{2}^{2} ,$$

where ${\mathbf{u}}$ and $\nu$ denote Lagrangian multiplier and penalty parameter, respectively. Equation (10) can be solved by three alternate iteration sub-problems. For simplicity, we do the following substitutions:

11 $$f_{\nu } ({\mathbf{x}},{\mathbf{v}},{\mathbf{u}}) = \frac{1}{2}||{\mathbf{y}} - {\mathbf{Ax}}||_{2}^{2} + \Re ({\mathbf{v}}) + \frac{\nu }{2}||{\mathbf{x}} - {\mathbf{v}} + {\mathbf{u}}||_{2}^{2} ,$$

then the alternate iteration subproblem is as follows:

12 $$\left\{ \begin{aligned} & {\mathbf{x}} \leftarrow \mathop {\min }\limits_{{\mathbf{x}}} f_{\nu } ({\mathbf{x}},{\mathbf{v}},{\mathbf{u}}) \hfill \\& {\mathbf{v}} \leftarrow \mathop {\min }\limits_{{\mathbf{v}}} f_{\nu } ({\mathbf{x}},{\mathbf{v}},{\mathbf{u}}) \hfill \\ & {\mathbf{u}} \leftarrow {\mathbf{u}} + {\mathbf{x}} - {\mathbf{v}} \hfill \\ \end{aligned} \right..$$

Alternate directions method of multipliers (ADMM) can be combined with TV [40] and dictionary learning [11] to complete MRI reconstruction together. When the ADMM algorithm is expanded into a network, different network versions can be constructed according to the learning situation of the network, for instance, image transformation has been replaced by the network for ADMM-net-I [41], ADMM-net-II [41] also learns data consistency except for image transformation.

## Deep learning with fully sampled k-space data

In the past few years, deep learning has achieved outstanding performance in the medical field, including biological magnetic resonance spectroscopy [42–46] and accelerated MRI [36, 39, 47–52]. MRI reconstruction based on deep learning can be roughly divided into two categories, data-driven and model-driven. The former uses the redundant information in the original input to learn the potential mapping relationship from input to output, including learning the mapping from zero-filed to artefact-free images [47] and the interpolation rules of k-space [49, 50].

For the sake of allowing the network to exploit the information of the imaging system, the researchers proposed physical model-driven deep learning methods [36, 39, 51, 52]. The network uses a fixed number of iterations to unroll the traditional optimization iterative algorithm, which not only achieves better reconstruction results but makes the network more interpretable. When there are a large number of training sample pairs, the supervised reconstruction can be expressed as follows,

13 $$\mathop {\arg \min }\limits_{\theta } \frac{1}{N}\sum\limits_{i = 1}^{N} {{\text{L}} ({\mathbf{x}}_{ref}^{i} ,f({\mathbf{y}}^{i} ,{\mathbf{A}}^{i} ,\theta ))} ,$$

where ${\mathbf{x}}_{ref}^{i}$ is the reference image of *i*th subject, ${\text{L}} ( \cdot , \cdot )$ is the loss function between the network output image and the reference image, and *N* is the number of fully sampled datasets in the training database. Let $f({\mathbf{y}}^{i} ,{\mathbf{A}}^{i} ,\theta )$ denotes output image of network for under-sampled k-space data ${\mathbf{y}}^{i}$ and measurement operator ${\mathbf{A}}^{i}$ of *i*th subject, where the network is parameterized by $\theta$. Equation (13) can be implemented using stochastic gradient descent (SGD) [53], whose basic form is

14 $$\theta^{k + 1} = \theta^{k} - \rho^{k} \frac{1}{N}\sum\limits_{i = 1}^{N} {\nabla {\text{L}} ({\mathbf{x}}_{ref}^{i} ,f({\mathbf{y}}^{i} ,{\mathbf{A}}^{i} ,\theta ))} ,$$

where $\rho^{k}$ is the gradient descent size. According to actual needs, it can change with the number of iterations, or be a constant. Due to the slow convergence of the basic SGD, researchers use other variants of SGD to speed up the convergence of the algorithm and avoid convergence to the saddle point [54–56]. Furthermore, unrolled network based on the traditional optimization algorithm is often used to improve the interpretability of the network and reconstruction quality [36, 39, 57, 58]. A comprehensive review of model-driven MRI deep learning reconstruction can be found in [7].

## Deep learning without fully sampled k-space data

**Fig. 1:**
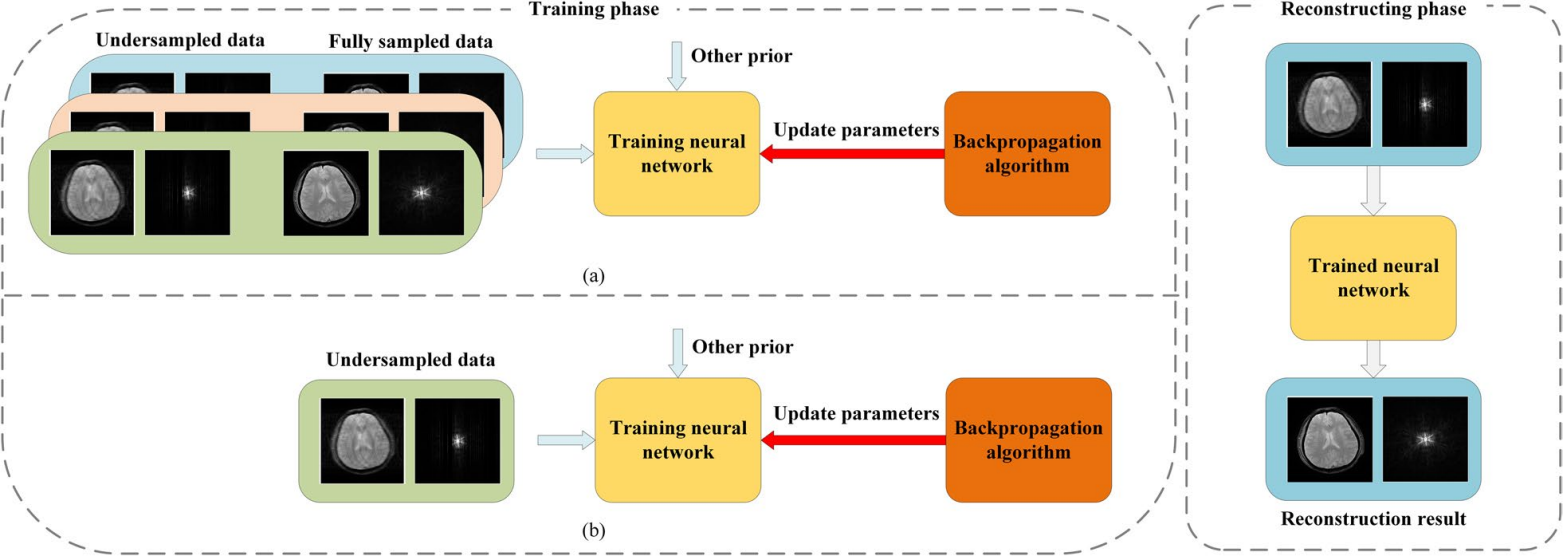
Flowchart of deep learning for MRI reconstruction with fully sampled data (a) and without fully sampled data (b). The difference between (a) and (b) is that (a) can train the network in a supervised manner. The network takes undersampled data and other prior as inputs and update parameters by backpropagation algorithms such as SGD and its variation. In reconstructing phase, the trained network can reconstruct high-quality images from the input

As discussed above, a network in a supervised manner can learn maps to complement the missing information in the input from fully sampled data. However, for the scenario without fully sampled data, it is difficult to find the optimal solution from infinite latent solutions without other information. For traditional optimization algorithms, the regularization term is usually manually pre-defined to obtain the optimal solution by compressing the solution space. Hence, how to better discover effective prior information is very important for deep learning without fully sampled k-space data, the flowchart is shown in Fig. 1.

Next, we will show the MRI reconstruction process in a deep learning manner from the perspective of a prior acquisition.

### Deep image prior

The experiment in [59] showed that only a generation network can still achieve good results in the absence of any other reference data, which illustrate that the convolutional neural network can replace the regularization term in Eq. (2) by capturing the low-level image implicit prior.

Yazdanpanah et al. [60] and Senouf et al. [61] apply this idea to MRI reconstruction, only use the zero-filled image as the input of the network, and then iteratively update the network parameters to approximate the k-space of the output image to the under-sampled k-space data. This idea was further extended to dynamic MRI by Jin et al. [62]. However, there are also obvious shortcomings, since it required well-designed network architectures and was easy to overfit to noise with the iteration gradually approaches the target as the optimization function is based on lossy data. Hence the regularization term was introduced to alleviate this situation [63]. Meanwhile, many works illustrate that unroll network based on the physical model can improve the interpretability of the network and reconstruction quality, and some of these traditional algorithms are briefly introduced in "Traditional methods for MRI reconstruction" section.

**Fig. 2:**
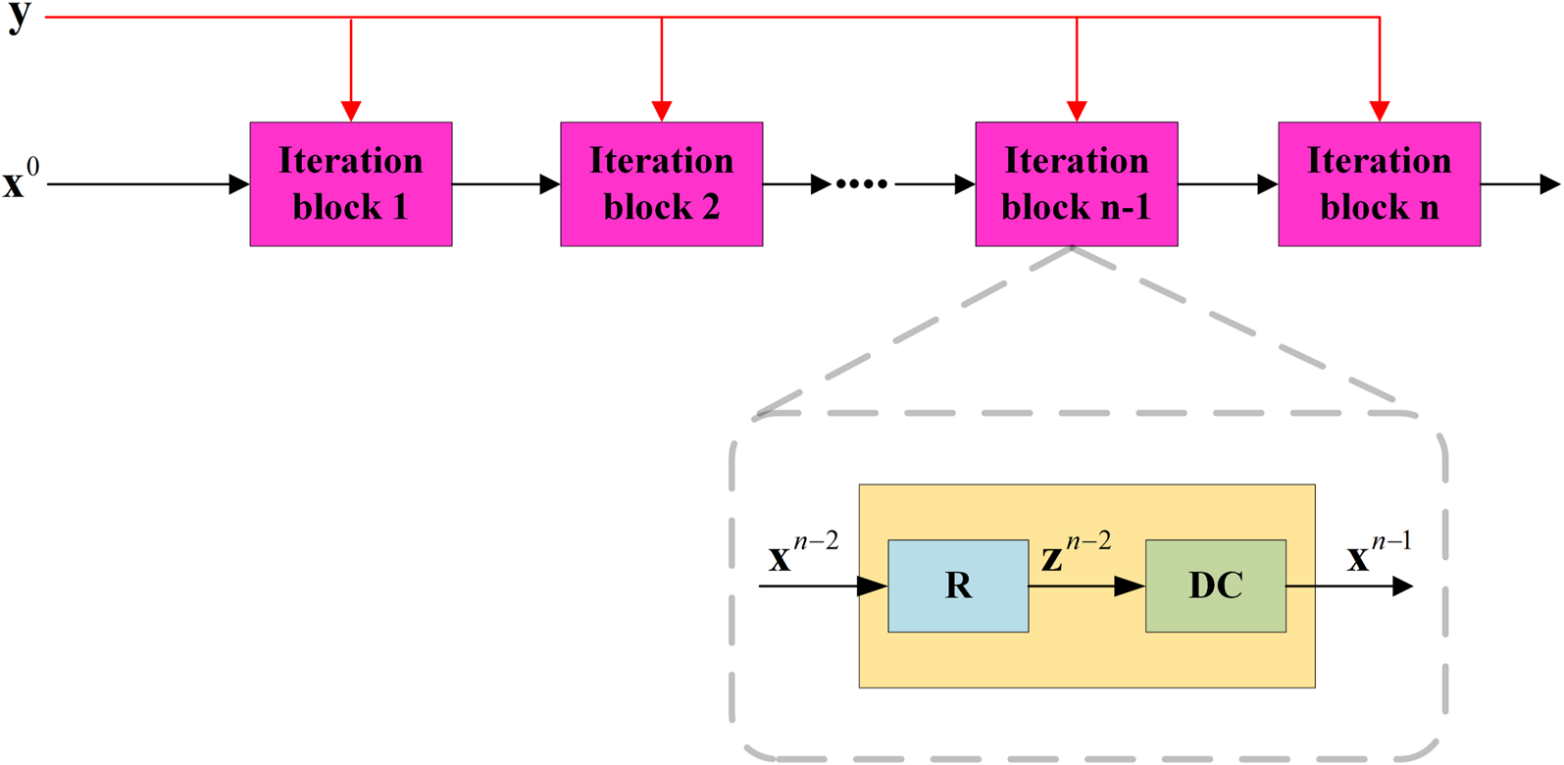
Unrolled network frame for VSQP. Here, each block consists of a regularization R and a data consistency (DC), which correspond to Eq. (3) and Eq. (4) respectively in VSQP

A. Wang et al. [64] proposed to combine a VQSP-based iterative network (as depicted in Fig. 2) with the high robustness of the classic iterative algorithm. The loss function adds a regularization term based on [60] as follows,

15 $$L({\mathbf{y}},\theta ) = \frac{1}{N}\sum\limits_{i = 1}^{N} {[||{\mathbf{A}}I_{\theta } ({\mathbf{y}}_{{\mathbf{i}}} ) - {\mathbf{y}}_{{\mathbf{i}}} ||_{2}^{2} + \Re (I_{\theta } ({\mathbf{y}}_{{\mathbf{i}}} ))]} ,$$

where $I_{\theta } ({\mathbf{y}}_{{\mathbf{i}}} )$ is an output of the network, the $\Re ( \cdot )$ is pre-defined such as TV. Although the addition of the $\Re ( \cdot )$ can ameliorate the situation in [60] that is easy to overfit to noise, it will encourage the solution to approach the characteristics of the pre-defined regularization term. The experimental performance revealed that the model in [64] was more robust than the supervised learning without using the above loss function, and had a better reconstruction utility compared with the classical method using the same loss function.

### Self-partition k-space data

**Fig. 3:**
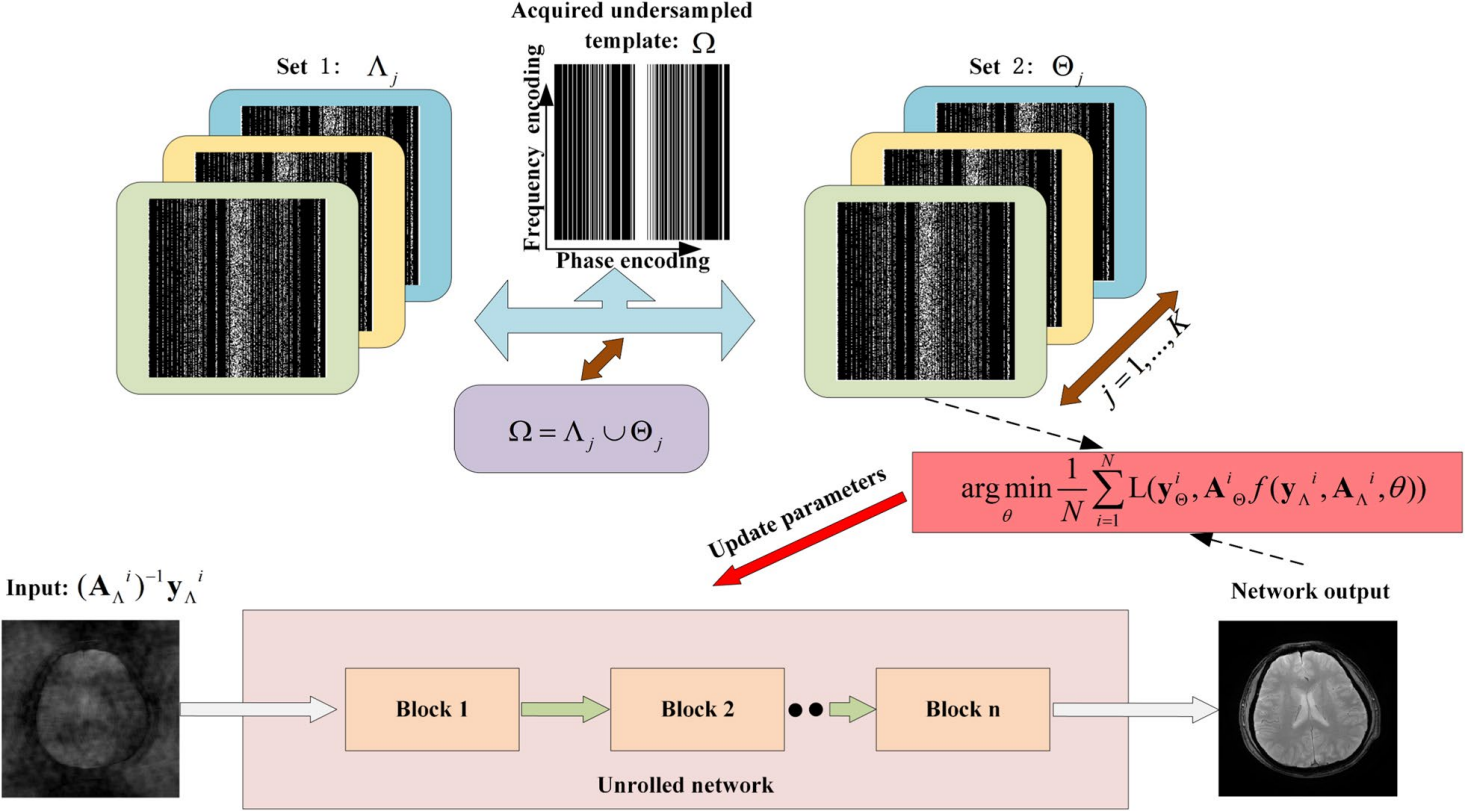
Image reconstruction with self-partition undersampled k-space data. Acquired undersampled k-space data $\Omega$ will be divided into two subsets satisfying $\Omega = \Lambda_{j} \cup \Theta_{j}$ before training network, where $j = 1,\ldots, {\text{K}}$ denoting the number of partitions for each scan, $\Lambda$ and $\Theta$ is used as input for training and to calculate the loss function separately. The network is unrolled based on the VSQP algorithm. This figure is reproduced following Fig. 1 in Ref. [65]

Yaman et al. [32] proposed to divide the measured under-sampling space $\Omega$ into two disjoint subsets satisfying $\Omega = \Lambda \cup \Theta$ to train VSQP -based unrolled reconstruction network, where $\Lambda$ was used as the network input for training and $\Theta$ was used to calculate the loss function, which could be called self-supervised learning via data under-sampling (SSDU). The experimental results showed that at a certain moderately acceleration rate the mentioned method can achieve comparable performance to supervised learning with fully sampled data and be better than traditional compressed sensing and parallel imaging. Since the under-sampled data needs to be divided, the information provided to the network for learning is further reduced, which will result in a poor network reconstruction performance at a high acceleration time. Thereby, Yaman et al. [65] further proposed a multi-mask method increasing the use of under-sampled data to improve the quality of reconstruction at higher acceleration rates. Here, the under-sampling data was divided into multiple disjoint subsets $\Omega = \Lambda_{j} \cup \Theta_{j}$ for $j$ = 1, …, K denoting the number of partitions for each scan. We can visualize the process through Fig. 3. The result illustrated that the multi-mask method outperforms SSDU at high acceleration rates. Even so, since the essence that partition will decrease information to the network can not be changed, acceleration rates are still limited. Furthermore, Hosseini et al. [66] tried to fine-tune the pre-trained reconstruction network in a scan-specific manner by using SSDU to reduce the risks of generalization to rare pathological conditions.

### k-space Information complement each other

The lack of fully sampled data motivates to study how to use the information available in lossy images [67]. The key problem that we can not use supervised learning for network training is that we do not have missing information as a label in each sample.

Inspired by Noise2Noise (N2N) [67], the artefact-contaminated dataset obtained by sampling the same object multiple times will supplement the information missing in each sample, which enables the training of the imaging priors. However, sampling multiple times for the same object violates the original intention of accelerating MRI reconstruction and not requiring fully sampled data, hence it has a little restriction on application scenarios.

Different body parts and regions of interest have unique complications, such as the liver that needs to hold the breath for imaging and cardiac that produce motion. Thereby, specific deep learning models need to be adapted for specific tasks [68, 69]. Gan et al. [70] use the middle adjacent layers having the most relevant brain regions in each object from open dataset OASIS-3 [71] to simulate multiple sampling of the same object. While the training data comes from the different breathing phases within the same slice of the liver in [72], which has obvious shortcomings for patients who do not have periodic breathing. Similarly, for organs such as cardiac that produce motion, Ke et al. [73] used a time-interleaved acquisition scheme to build a series of fully encoded data as reference images for network training by merging the k-space of several adjacent frames along the time dimension. The remaining part will explain more details.

Gan et al. [70] proposed to train two networks simultaneously, one was used for reconstruction by utilizing information supplement between different samples, and another to register the image as the object may have moved in the actual scanning process. The experimental results showed that the method was superior to the unregistered Noise2Noise method and the traditional total variation (TV) method in terms of sharpness, contrast and de-artefacts.

Additionally, experiments have shown that converting advanced denoising devices into regularization items can achieve good results [63, 74, 75], regularization by denoising (RED) [74] and the plug-and-play-prior (PnP) [76] are two common skills. Meanwhile, the deep network can be flexible to extract useful information from the data set compared with handcrafted prior. Hence Liu et al. [72] proposed to pre-train a de-artefact network as imaging prior through information complement between under-sampled data, the regularization function used the basic framework of RED, where the denoiser was replaced by the pre-train network $I_{\theta } ({\mathbf{x}})$ in the following form.

16 $$\Re ({\mathbf{x}}) = \frac{\tau }{2}{\mathbf{x}}^{T} ({\mathbf{x}} - I_{\theta } ({\mathbf{x}}))$$

where $\tau$ is a regularization parameter. Bring Eq. (16) into Eq. (2), then iteratively update ${\mathbf{x}}$ by using the simplest gradient descent algorithm for Eq. (2). Due to the use of data fidelity information, compared to the results obtained directly through $I_{\theta } ({\mathbf{x}})$, the proposed method reconstruction results on liver data were better and outperformed traditional iterative algorithms, such as k-t SLR. Moreover, we can use more advanced traditional iterative algorithms to update ${\mathbf{x}}$ such as ADMM and so on.

**Fig. 4:**
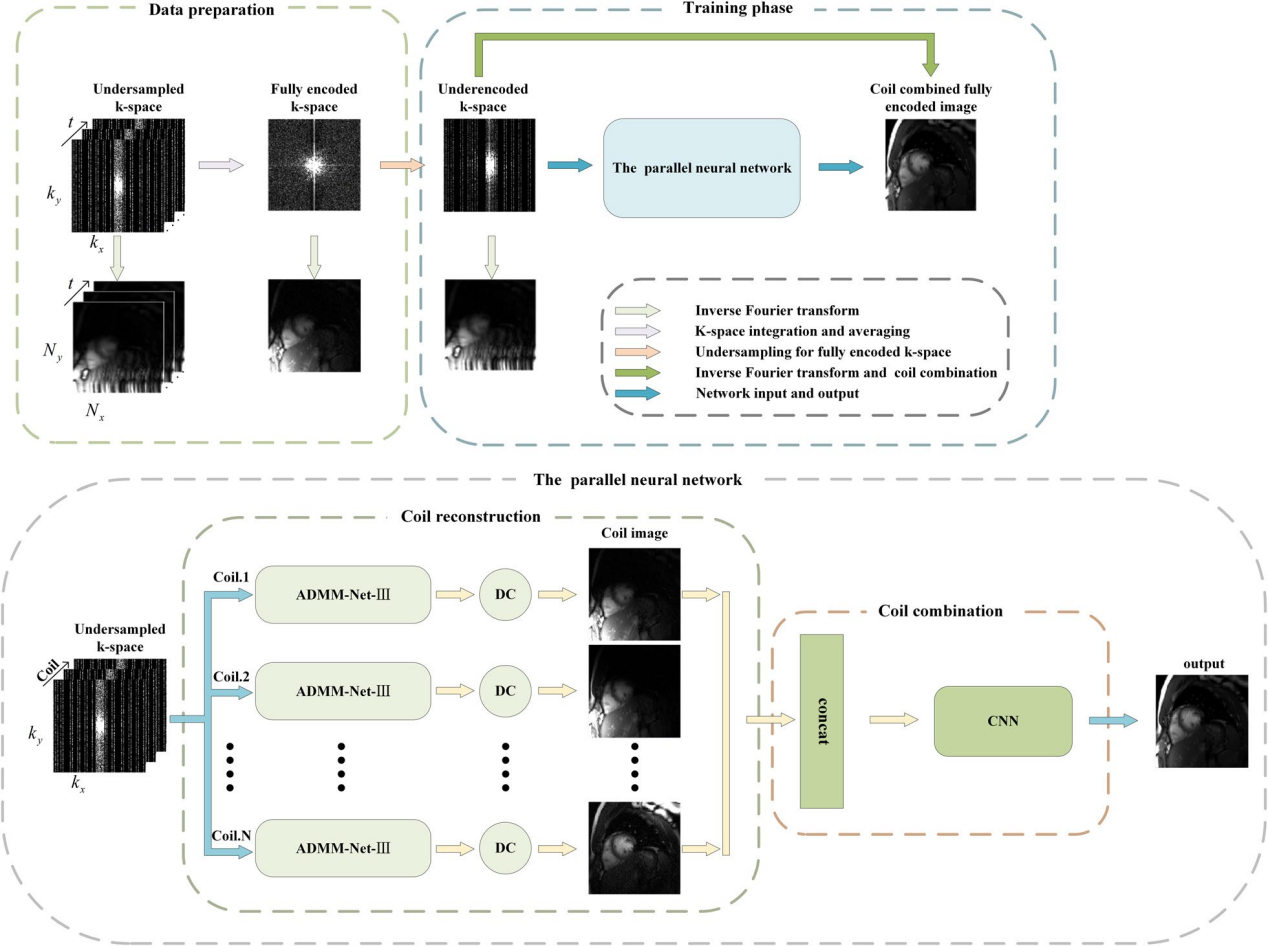
The structure diagram in [73]. In data preparation, the fully encoded k-space is obtained by k-space integration and averaging of multiple frames in a time-interleaved sampling manner, then which is undersampled with a designed sampling mask and performs some operations including inverse Fourier transform and coil combination to get input and output data pairs separately. The parallel neural network consists of coil reconstruction and coil combination, we can refer to [41] for more details about ADMM-Net-III. This figure is reproduced following Fig. 1 and Fig. 3 in Ref. [73]

In [73], Ke et al. averaged all acquired frames to improve the signal-to-noise ratio (SNR) and relieve memory pressure. It should be noted that the merge operation only occurs in the training sample synthesis stage, and it is not required in the subsequent testing stage. Therefore, the reconstructed image will not result in a lower temporal resolution. Concretely, a network is established to learn the correlation between the coils instead of obtaining through ESPIRIT [77], the physical model-based ADMM-Net-III [41] was used as the reconstruction network. The method structure diagram is shown in Fig. 4. The experimental results showed that the reconstruction quality was better than conventional reconstruction methods, such as k-t SLR [13], L + S [78], KLR [79], etc., and the reconstruction time was shorter [73]. Nevertheless, as the breathing pattern is inconsistent with different people, the generalization ability of the model will be affected.

### Assuming known probability distribution as prior

Generative Adversarial Networks (GAN) [80] has shown strong advantages in unsupervised learning and can estimate the basic data distribution while obtaining higher image quality [81, 82]. In principle, the purpose of adversarial training is to approximate, in terms of distances, the probability distribution of the label set. Hence, there is no need for the ground truth corresponding to the input. Nevertheless, the real probability distribution between the generated output and the input of the discriminator will directly affect the utility of the final generator output as both of them approximating when the generator and discriminator obtaining equilibrium.

**Fig. 5:**
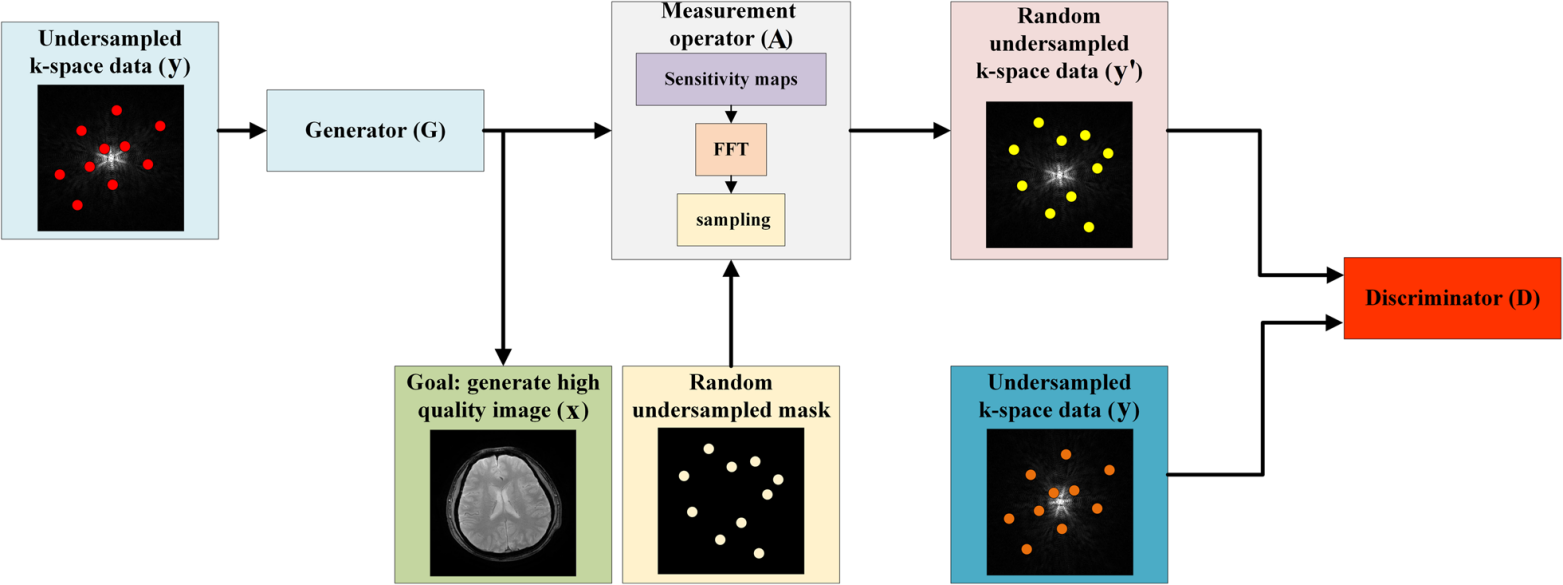
Unsupervised GAN learning system. The input and output of the generator is measurement complex-valued k-space data and two-dimensional image, then the output of generator performs forward measurement operation including a random undersampled mask to get simulation undersampled k-space data, finally, discriminator tries to distinguish between simulation data and measurement data. This figure is reproduced following Fig. 1 in Ref. [83]

Cole et al. [83] proposed to build an ISTA-based GAN network for MRI reconstruction in the absence of fully sampled data. The network framework shown in Fig. 5 is based on the assumption that the randomly undersampled k-space ${\mathbf{y^{\prime}}}$ has the approximate distribution as the initially obtained measured k-space ${\mathbf{y}}$, where ${\mathbf{y}}\prime$ is obtained by performing forward measurement operation including random undersampled mask on the output of the generator. Hence, since the distribution ${\mathbf{A}}$ is known, the true underlying distribution of ${\mathbf{x}}$ can be uniquely determined ${\mathbf{y}}\prime$ [84].

Meanwhile, Wasserstein distance is continuous and differentiable compared with Jensen-Shannon to serve as the measure of the distance between two probability distributions [85], and it is variation WGAN-GP which has proven to have the best convergence performance [86] was selected as the loss function of the network here. Ultimately, the experiment was carried out on dynamic contrast-enhanced (DCE) data and knee data, and the results showed that the reconstruction quality has competed to supervised counterpart and better than CS.

Besides, experiments have proved that image style transfer tasks do not need ground-truth can be finished by unpaired training with only adversarial training [87], which is based on the assumption that there is a potential distribution relationship between unpaired samples and try to let the network learn this relationship. Hence, Lei et al. [88] suggested 2D images that are easier to obtain can be used as training labels for DCE images to train a PGD-based GAN network, Sim et al. incorporate the cycle consistent generative adversarial network (cycleGAN) [87] and forward physics in MRI using optimal driven theory to complete unpaired samples training [89]. For this type of method, the degree of joint probability distribution between unpaired samples will directly affect the experimental results. Thereby, the experimental results can be predicted to be no better than CS because the unpaired samples are completely disjoint in [88].

In addition to the mentioned methods, there are other ways to solve the problem of network training without fully sampled images. For example, traditional parallel compressed sensing imaging can be used as the true label for network training [90], but the final reconstruction quality of the network will not be significantly better than traditional parallel compressed sensing [83].

## Conclusions

In summary, deep learning can complete related tasks by learning an implicit mapping relationship. Although the learning process cannot be explained in detail, many experimental results show that deep learning is effective and feasible. In normal conditions, the performance of supervised learning is better than that of network learning without labels. But in many scenarios, it is very difficult and infeasible to obtain labels, which seriously hinders the application of supervised learning, and also highlights the importance of deep learning without ground truth. Despite the reviewed methods are applied to MRI reconstruction, it can also be extended to other areas where it is difficult to obtain real data, such as dynamic positron emission tomography (PET) [91] or computed tomography (CT) [92].

While deep learning shows strong learning capabilities, it has also been criticized for its poor interpretability. Accordingly, the theoretical research of deep learning has become a new hot research direction. Researchers try to analyze the effective network structure from different angles to guide the construction of new networks, such as differential equations [93] and matrix decomposition [94].

Nevertheless, exploring the effective prior information in the lossy data and the inherent characteristics of the network is still a direction that needs to be studied. In addition, an appropriate sampling strategy for a specific body part is important for deep learning reconstruction performance. Typically, fast coronary imaging usually uses the spiral under-sampling scheme [95] and some studies try to learn sampling strategy and reconstruction at the same time through the network [96].

Fortunately, inspired by the unfolding network based on the physical model. As traditional iterative algorithms and deep learning have their advantages, the former is computationally complex but has strong guidance significance, and the latter has the advantages of real-time imaging and powerful learning capabilities. We may anticipate that the deeper integration of traditional methods and deep learning, which will not only guide the construction of networks to bring more interpretability but also more importantly, can obtain better results. Additionally, information complementarity between multi-contrast images may be used as priors to participate in reconstruction.

## Data Availability

No datasets were generated or analyzed during the current study in this review.

## Abbreviations

ADMM: Alternate directions method of multipliers
CG: Conjugate gradient
CT: Computed tomography
CS: Compressed sensing
DC: Data consistency
DCE: Dynamic contrast-enhanced
FFT: Fast Fourier transform
GAN: Generative adversarial networks
ISTA: Iterative shrinkage-thresholding algorithm
MRI: Magnetic resonance imaging
MSSIM: Mean structure similarity index measure
N2N: Noise2Noise
PSNR: Peak signal-to-noise ratio
PET: Positron emission tomography
pFISTA: Projected iterative soft-thresholding algorithm
PGD: Proximal gradient descent
PnP: Plug-and-play-prior
RED: Regularization by denoising
SGD: Stochastic gradient descent
SSDU: Self-supervised learning via data under-sampling
TV: Total variation
VSQP: Variable-splitting with the quadratic penalty
